# The multifaceted role of FAM13A in pulmonary diseases

**DOI:** 10.3389/fgene.2026.1820547

**Published:** 2026-05-26

**Authors:** Zhengqiang Luo, Keyan Lu, Mingqian Lai, Sixuan Liang, Guoda Ma, Yajun Wang

**Affiliations:** 1 Institute of Pediatrics, Shunde Women and Children’s Hospital, Guangdong Medical University, Foshan, China; 2 Institute of Pediatric Hemato-oncology, Shunde Women and Children’s Hospital, Guangdong Medical University, Foshan, China

**Keywords:** asthma, COPD, *FAM13A*, genetic susceptibility, molecular mechanisms, pulmonary diseases

## Abstract

*FAM13A*, a lung-enriched gene encoding a protein with a characteristic RhoGAP domain, is increasingly recognized for its pleiotropic roles across multiple lung diseases, including chronic obstructive pulmonary disease (COPD), pulmonary fibrosis (PF), asthma, and lung cancer. Through modulation of Rho and Wnt/β-catenin signaling, *FAM13A* regulates key cellular processes such as epithelial barrier maintenance, immune homeostasis, and cell-cycle regulation. Notably, *FAM13A* exhibits context-dependent duality, associated with tissue destruction in COPD while linked to mitigated fibrotic remodeling in PF. In addition, the complexity introduced by multiple splice variants, interspecies expression differences, and environmental dependence poses significant challenges for further mechanistic studies of *FAM13A*. By emphasizing the opposing roles of *FAM13A* in COPD versus PF, the non-linear relationships linking single-nucleotide polymorphisms (SNPs), gene expression, signaling pathways, and disease phenotypes, as well as the combined influence of isoform diversity, species differences, and environmental exposures on functional outcomes, this review integrates current genetic, molecular, and functional evidence to provide a mechanistic framework for understanding *FAM13A*’s roles in pulmonary diseases and refines current paradigms with implications for future research and precision medicine.

## 1 Introduction

Pulmonary diseases represent a major global health burden and leading cause of disability and mortality worldwide. Their pathogenesis involves complex interactions between genetic susceptibility, environmental exposures, and immune responses. Although chronic obstructive pulmonary disease (COPD), asthma, pulmonary fibrosis (PF), and lung cancer manifest distinct clinical phenotypes, they share core pathological mechanisms characterized by chronic inflammation, oxidative stress, and immune dysregulation (Racanelli et al., 2018; Lamers and Haagmans, 2022). At the molecular level, numerous signaling pathways have been identified as critical regulators of pulmonary disease initiation and progression (Mishra et al., 2018). Genetic factors likewise exert profound effects on disease susceptibility, clinical variability, and therapeutic responses (Young et al., 2011). Among the genes identified through comprehensive genetic analyses, *FAM13A* (family with sequence similarity 13 member A; OMIM 613299) has emerged as a pleiotropic determinant across multiple pulmonary diseases, underscoring the shared genetic basis of respiratory pathogenesis.

*FAM13A* was first identified as a major genetic locus influencing COPD susceptibility, as revealed by large-scale genome-wide association studies (GWAS) (Young et al., 2011). Expression analyses reveal that FAM13A shows highest expression in adult lung tissue and bronchial epithelial cells, with lower levels in fetal and aged lungs (Miller et al., 2016; Chen et al., 2023). Subsequent investigations have established associations between FAM13A and other pulmonary diseases including asthma (Accordini et al., 2023), PF (Hirano et al., 2017), and lung cancer (Ziółkowska-Suchanek et al., 2017). Current evidence indicates that FAM13A modulates critical pathological processes through regulation of cellular signaling pathways, maintenance of epithelial barrier function, and modulation of immune responses. Despite these advances, the molecular mechanisms underlying FAM13A’s functions remain incompletely characterized, particularly its context-dependent effects in different disease states such as COPD and PF (Hobbs et al., 2017).

## 2 Search strategy

This review is non-systematic in nature. To identify literature on the role of FAM13A in respiratory diseases, we searched the PubMed and Web of Science databases in December 2025 using the search term “FAM13A″ without any time restrictions. The initial search yielded 218 records.

We manually screened the titles and abstracts of all retrieved records for relevance to the topic of this review. Priority for inclusion was given to studies that were: (1) functional experiments (*in vitro* and/or *in vivo*) involving FAM13A; (2) directly related to lung diseases; and (3) investigations of *FAM13A-AS1* (the FAM13A-associated long non-coding RNA). For potentially relevant studies meeting these priorities, the full text was reviewed to confirm eligibility for inclusion. Additionally, we manually searched the reference lists of the included articles to identify any further relevant studies.

As this is a narrative review, no formal quality assessment or quantitative meta-analysis was performed.

### 2.1 Part 1: structural and functional features of FAM13A

#### 2.1.1 Gene structure and isoforms

The *FAM13A* gene (also designated *FAM13A1*, *KIAA0914*, and *ARHGAP48*) was originally identified through studies of quantitative trait loci for milk production in cattle and subsequently cloned in 2004 (Cohen et al., 2004). This gene maps to chromosome 4q22 in humans, spans 331.23 kb, and comprises 32 exons. Five principal splice isoforms have been characterized in humans: *FAM13A*-a (NM_014883.4), *FAM13A*-b (NM_001015045.3), *FAM13A*-c (NM_001265578.2), *FAM13A*-d (NM_001265578.2), and *FAM13A*-e (NM_001265580.2), each exhibiting distinct tissue-specific expression profiles (Table 1).

**TABLE 1:** *FAM13A* gene Isoforms Listed in the NCBI Database (updated August 2025).

Fam13A isoform	mRNA accession	mRNA size (bp)	Protein accession	Protein size (aa)
Curated Transcripts (NCBI)				
Isoform a	NM_014883.4	5,866	NP_055698.2	1,023
Isoform b	NM_001015045.3	4,895	NP_001015045.1	697
Isoform c	NM_001265578.2	4,853	NP_001252507.1	683
Isoform d	NM_001265579.2	4,811	NP_001252508.1	669
Isoform e	NM_001265580.2	4,811	NP_001252509.1	669
No-curated Transcripts (NCBI)				
x1	XM_011531516.2	5,737	XP_011529818.1	1,023
x2	XM_047449479.1	5,695	XP_047305435.1	1,009
x3	XM_011531517.3	5,782	XP_011529819.1	995
x4	XM_005262683.4	5,782	XP_005262740.1	995
x5	XM_017007624.3	5,740	XP_016863113.1	981
x6	XM_047449480.1	5,740	XP_047305436.1	981
x7	XM_017007625.1	5,495	XP_016863114.1	968
x8	XM_047449481.1	5,569	XP_047305437.1	967
x9	XM_047449482.1	5,656	XP_047305438.1	953
x10	XM_017007626.1	5,129	XP_016863115.1	846
x11	XM_011531518.2	5,436	XP_011529820.1	837
x11	XM_047449483.1	5,352	XP_047305439.1	837
x11	XM_011531519	5,480	XP_011529821.1	837
x12	XM_006714057.4	5,153	XP_006714120.1	834
x13	XM_047449485.1	4,979	XP_047305441.1	751
x13	XM_047449484.1	5,064	XP_047305440.1	751
x14	XM_017007633.3	4,980	XP_016863122.1	702
x15	XM_017007634.3	4,769	XP_016863123.1	655
x16	XM_047449487.1	4,727	XP_047305443.1	641

**FIGURE 1:**
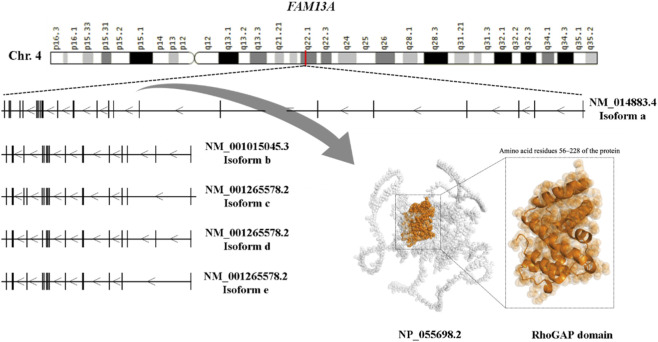
Isoforms of the *FAM13A*.FAM13A exists as multiple splice isoforms. The full-length isoform a contains the complete RhoGAP domain, suggesting complex transcriptional regulation and functional diversity. Upper panel: exon composition of the five validated isoforms (a-e). Lower panel: AlphaFold-predicted 3D structure of isoform a, with the RhoGAP domain highlighted. Isoforms depicted: a (1,023 amino acids, containing the RhoGAP domain), b (697 amino acids, containing two coiled-coil domains), and c-e (incompletely characterized). RhoGAP: Rho GTPase-activating protein. Take-home message: FAM13A exists as multiple splice isoforms; the full-length isoform a contains the complete RhoGAP domain, suggesting complex transcriptional regulation and functional diversity.

The canonical *FAM13A*-a isoform contains 24 exons and encodes a 1,023-amino acid protein (∼116.9 kDa) featuring an N-terminal RhoGAP domain flanked by central and C-terminal coiled-coil structural motifs. This isoform demonstrates highest expression in pulmonary, thymic, renal, pancreatic, and hepatic tissues (Yang et al., 2013). In contrast, the *FAM13A*-b variant comprises 18 exons, diverges in the 5′UTR sequence, and utilizes an alternative translation initiation site, yielding a 697-amino acid polypeptide (∼80.2 kDa) containing two coiled-coil domains with predominant expression in skeletal muscle, thymus, brain, and pulmonary tissue (Corvol et al., 2014). The remaining three documented isoforms remain inadequately characterized (Figure 1). Notably, *in silico* analyses predict 19 additional transcript variants that remain to be experimentally validated, suggesting substantial unresolved complexity in the gene’s regulatory landscape.

#### 2.1.2 RhoGAP domain and signaling regulation

**FIGURE 2:**
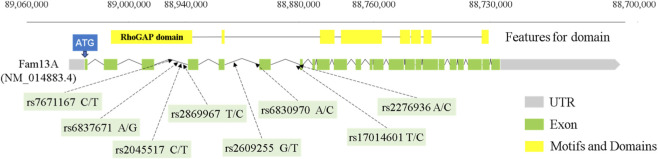
Genomic structure and functional domains of FAM13A. Shown are the genomic structure of the long isoform (FAM13A-a), its protein functional domains (N-terminal RhoGAP domain and coiled-coil motifs), and four SNP sites (rs7671167, rs6837671, rs2045517, rs2869967) located within the RhoGAP domain. This isoform shows the highest expression in lung tissue and contains the complete functional domain; these SNPs are significantly associated with COPD, pulmonary fibrosis, and other diseases in multiple GWAS. RhoGAP: Rho GTPase-activating protein; SNP: single-nucleotide polymorphism. Take-home message: Multiple disease-associated SNPs are located within the RhoGAP domain, indicating that this domain is a key functional region through which FAM13A modulates disease risk.

The FAM13A protein primarily localizes to the cytoplasm, with additional distributions observed in nucleoli, nuclear bodies, and cell junctions within specific cell types (UniProt O94988; Human Protein Atlas ENSG00000138640). Despite not being a classical transmembrane protein, its subcellular localization pattern suggests involvement in cellular signal regulation. The protein’s most prominent structural feature is an N-terminal RhoGAP-like domain (encoded by exons 2–5) that, despite lacking canonical GTPase-activating function, modulates small GTPase activities including RhoA and Rac1, thereby influencing cytoskeletal dynamics, cell polarity, adhesion, and migration (Ridley, 2001; Tcherkezian and Lamarche-Vane, 2007) (Figure 2).

Notably, Rho signaling plays essential roles in maintaining pulmonary endothelial barrier integrity and epithelial homeostasis. Dysregulated activation of this pathway drives barrier breakdown, inflammatory responses, and tissue remodeling - central pathogenic mechanisms associated with COPD, idiopathic pulmonary fibrosis (IPF), and small cell lung cancer (Young et al., 2011; Duluc and Wojciak-Stothard, 2014). Furthermore, FAM13A functions as a negative regulator of Wnt/β-catenin signaling through promoting β-catenin degradation and restricting its nuclear translocation, thereby suppressing epithelial cell proliferation, apoptosis, and fibrotic activation (Jiang et al., 2016).

Evidence from extrapulmonary systems further supports FAM13A’s role in signaling regulation. In metabolic tissues, FAM13A enhances insulin signaling in adipocytes by stabilizing IRS1, thereby regulating fat distribution and metabolic homeostasis (Fathzadeh et al., 2020; Grygiel-Górniak et al., 2023; Wardhana et al., 2018). These findings suggest FAM13A’s may be broadly involved in cellular metabolism and stress responses, supporting its potential functions in pulmonary homeostasis, oxidative stress management, and inflammatory regulation. Additionally, FAM13A displays tumor-modulatory activities in various solid malignancies, with its RhoGAP domain specifically implicated in governing tumor cell motility in non-small cell lung cancer (NSCLC) (Wang et al., 2022; Qiu et al., 2022; Hsieh et al., 2023; Eisenhut et al., 2017).

### 2.2 Part 2. *FAM13A* in pulmonary function

#### 2.2.1 Genetic variants in pulmonary function

GWAS have identified *FAM13A* as a susceptibility locus associated with impaired pulmonary function. Multiple single-nucleotide polymorphisms (SNPs) within this locus exhibit significant associations with key spirometric indices, including forced expiratory volume in 1 s (FEV_1_), the FEV_1_/forced vital capacity (FEV_1_/FVC) ratio, and diffusing capacity for carbon monoxide (DLCO) (Accordini et al., 2023; Hobbs et al., 2017; Lee et al., 2021; Pillai et al., 2010; Van der plaat et al., 2017; Lutz et al., 2015).

In Korean cohorts, a haplotype block in strong linkage disequilibrium (*r* ^2^ > 0.8) containing SNPs rs2609264, rs2609261, and rs2609260 shows a significant association with accelerated lung function decline. Notably, this genetic effect exhibits a multiplicative interaction with smoking history (Kim et al., 2014a; Kim et al., 2015). Subsequent exome array analyses further identified rs7671167 as independently associated with lung function impairment (Lee et al., 2021). In addition, adjacent variants rs10007590 and rs2869966 exhibit respective associations with reduced FEV_1_ levels and decreased FEV_1_/FVC ratio (Pillai et al., 2010).

Evidence from Japanese populations further illustrates the genetic complexity of this locus. A Japanese cohort study revealed a complex dual role for rs2609255, where the major T allele accelerated DLCO decline in established PF patients (β = −7.2) yet appeared to confer protection against PF development in the general population (OR = 1.78 for risk-associated G allele) (Hirano et al., 2017; Wang et al., 2021; Jönsson et al., 2022). Moreover, epigenetic regulation through DNA methylation near the *FAM13A* locus has been shown to fetal lung development and pulmonary function (Kerkhof et al., 2014; Gruzieva et al., 2019). These findings collectively position FAM13A as a risk region harboring multiple independent signals whose effects may vary by disease status, developmental stage, and intricate interactions between cellular and environmental factors.

#### 2.2.2 Gene–environment interplay shaping *FAM13A*-associated pulmonary function

*FAM13A* is a gene closely linked to environmental exposures. Under physiological conditions, human FAM13A gene is predominantly highly expressed in ciliated cells of the airway epithelium (Vieira Braga et al., 2019). Under conditions without environmental threats, FAM13A is believed to help maintain the health and function of lung tissue (Kerkhof et al., 2014). Consistently, overexpression of the FAM13A gene in senescent 16HBE cells reduces the production of mitochondrial reactive oxygen species (ROS), demonstrating a protective effect against oxidative stress (Chen et al., 2023). However, under adverse environmental exposures, FAM13A shifts from a protective to a detrimental role in lung function (Kerkhof et al., 2014). For instance, prenatal exposure to particulate matter (PM10) induces DNA methylation near the FAM13A locus, impairing fetal lung development and function (Kerkhof et al., 2014; Gruzieva et al., 2019). Cigarette smoke (CS)-exposed bronchial epithelial cells, overexpression of FAM13A activates SIRT1, leading to upregulation of carnitine palmitoyl transferase 1A (CPT1A), enhancing fatty acid oxidation (FAO) and promoting reactive oxygen species (ROS) accumulation. These processes trigger alveolar epithelial cell death, exacerbate alveolar wall destruction and emphysema (Hawkins and Mora, 2017; Jiang et al., 2017).

**FIGURE 3:**
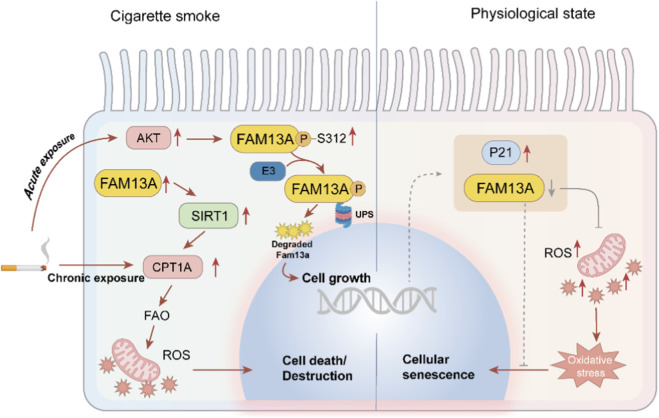
Context-dependent roles of FAM13A under senescence and CS–induced conditions. FAM13A acts differently under physiological vs. smoke exposure. Left panel: After acute exposure, AKT phosphorylates FAM13A at S312, recognized by E3, triggering ubiquitin-dependent degradation and promoting repair; after chronic exposure, FAM13A interacts with SIRT1, enhancing CPT1A/FAO/ROS and promoting cell death. Right panel: During physiological aging, FAM13A declines, increasing p21 and mitochondrial ROS, promoting senescence; FAM13A overexpression reduces p21 and ROS. FAO: fatty acid oxidation; ROS: reactive oxygen species; E3: CULLIN4A E3 ubiquitin ligase; AKT: protein kinase B. Take-home message: FAM13A is context-dependent — repair upon acute exposure, injury upon chronic exposure, senescence during aging.

*In vitro* studies demonstrate that lung tissue injury triggers ubiquitin-mediated degradation of FAM13A, which facilitates proliferative repair by lung epithelial progenitor cells (Gong et al., 2023) and exerts anti-inflammatory effects (Chen et al., 2021). In murine models of smoke-induced airway injury, FAM13A deficiency enhances the repair and regenerative capacity of alveolar type II cells, thereby mitigating alveolar destruction (Lin et al. 2021). Furthermore, studies in CS-exposed mice reveal that FAM13A forms a dynamic protein complex with PP2Ab and β-catenin, promoting β-catenin phosphorylation and degradation. This interaction suppresses β-catenin signaling, directly inhibiting the repair and regeneration of alveolar and airway epithelial cells (Jiang et al., 2016). In parallel, FAM13A also suppresses TGF-β2 transmembrane transport, thereby indirectly promoting lung injury progression (Gong et al., 2021) (Figure 3).

These observations highlight a context-dependent regulatory framework in which FAM13A modulates the temporal and spatial dynamics of lung repair. The gene’s divergent effects across injury stages suggest that its function is not fixed but continuously recalibrated in response to microenvironmental cues.

### 2.3 Part 3. genetic variations and functional implications

#### 2.3.1 Pleiotropic effects across pulmonary diseases

*FAM13A* polymorphisms demonstrate significant pleiotropy and complexity in their associations with respiratory disease risk, with effect sizes and directions varying markedly across different diseases and ethnic populations. The NCBI dbSNP database documents approximately 127,779 genetic variants in the *FAM13A* locus (accessed October 2025), though only a limited number have been functionally characterized or linked to pulmonary phenotypes—such as rs7671167 (Young et al., 2011; Ding et al., 2015; Cho et al., 2010; Xie et al., 2015; Wang et al., 2013; Arja et al., 2014; Kim et al., 2014b; Lee et al., 2014a), rs2609255 (Wang et al., 2021; Jönsson et al., 2022; Hu et al., 2023; Singh et al., 2024; Wang et al., 2018; Higuchi et al., 2023), rs2869967 (Lee et al., 2014a; Guo et al., 2011; Hancock et al., 2010; Li et al., 2011), rs6838671 (Lutz et al., 2015; El et al., 2021; Sakornsakolpat et al., 2019), rs17014,601 (Zhang et al., 2018; Pham et al., 2023), rs2045517 (Lee et al., 2014a; Lamontagne et al., 2013), rs2276936 (Accordini et al., 2016; Lin et al., 2020), rs6830970 (Wang et al., 2013; Hancock et al., 2010) (Table 2).

**TABLE 2:** Association between different SNPs of the *FAM13A* and diseases.

Site	Location	Exon/Intron	Disease	Impact	References
rs7671167	chr4:88962828	Intron:C>G/C>T	COPD/Lung Function/NSCLC	The ‘T' allele increases the risk of developing COPD and is associated with a decline in FEV_1_ and the FEV_1_/FVC ratio in lung function	Young et al. (2011), Ding et al. (2015), Cho et al. (2010), Xie et al. (2015), Wang et al. (2013), Arja et al. (2014), Kim et al. (2014b), Lee et al. (2014a)
rs2609255	chr4:88890044	Intron:G>T	ILD/Silicosis/IPF/RA-ILD/RA-PF	The ‘G' allele confers an increased risk of ILD/RA-ILD/RA-PF/Silicosis	Wang et al. (2021), Jönsson et al. (2022), Hu et al. (2023), Singh et al. (2024), Wang et al. (2018), Higuchi et al. (2023)
rs2869967	chr4:88948181	Intron:T>C/T>G	COPD/Asthma/Pulmonary Function	The ‘C' allele increases COPD risk and interacts with other genes to affect lung function in asthma	Lee et al. (2014a), Guo et al. (2011), Hancock et al. (2010), Li et al. (2011)
rs6837671	chr4:88951941	Intron:A>G	COPD/Pulmonary Function	The ‘G' allele is associated with reduced DLCO and increases COPD risk, independently or synergistically with low vitamin D	Lutz et al. (2015), El et al. (2021), Sakornsakolpat et al. (2019)
rs17014601	chr4:88809748	Intron:T>C	COPD	The ‘C' allele elevates COPD risk	Zhang et al. (2018), Pham et al. (2023)
rs2045517	chr4:88949813	Intron:C>T	COPD	The ‘T' allele is positively correlated with COPD risk through its association with increased FAM13A expression	Lee et al. (2014a), Lamontagne et al. (2013)
rs2276936	chr4:88805132	Intron:A>C/A>G	Pulmonary Function/FLD	Moderately correlates with lung function, improves insulin sensitivity, prevents fatty liver	Accordini et al. (2016), Lin et al. (2020)
rs6830970	chr4:88855930	Intron:A>C/A>G/A>T	COPD/Pulmonary Function	The AA and AG genotypes are associated with non-smoking and ex-smoking COPD patients, respectively, with AG also linked to higher FEV_1_/FVC	Wang et al. (2013), Hancock et al. (2010)

Additional population-specific associations have been established for other *FAM13A* variants. The rs1903003 polymorphism, maintained in strong linkage disequilibrium with rs7671167, shows reproducible association with COPD in European and Hispanic populations (Kim et al., 2014a; Ding et al., 2015). Similarly, the C allele of rs2869967 is associated with COPD risk determinant in Han Chinese (OR = 2.414) (Young et al., 2011; Cho et al., 2010; Xie et al., 2015; Guo et al., 2011; Lee et al., 2014b). Whereas the rs6837671 G allele is associates with moderate risk elevation in Middle Eastern populations (OR = 1.12). This effect is substantially potentiated through interaction with hypovitaminosis D, resulting in a 3.35-fold risk amplification (Young et al., 2011; EL et al., 2021).

The G allele of rs2609255 is an established risk factor associated with IPF, particularly in Asian and Hispanic populations (OR = 1.47–1.81), though this association is absent in Europeans (Hirano et al., 2017; Hu et al., 2023; Singh et al., 2024; Guzmán-Vargas et al., 2021). This allele also correlates with silicosis risk in Chinese populations (OR = 1.71) (Wang et al., 2018). For asthma, the TT/TC genotypes of rs987314 are significantly associate with elevated fractional exhaled nitric oxide (FeNO) levels, suggesting potential involvement in airway inflammation regulation (Accordini et al., 2023).

These findings collectively indicate that *FAM13A* genetic variants transcend single-disease boundaries, exerting heterogeneous effects on susceptibility and severity across pulmonary diseases, including both pleiotropic and paradoxical manifestations.

#### 2.3.2 How SNPs shape phenotypic heterogeneity

The mechanistic link between non-coding region *FAM13A* SNPs and phenotypic heterogeneity variation remains a subject of active investigation. Current evidence indicates that these effects are primarily mediated by allele-specific regulation of gene expression and interactions with cellular stressors.

The rs7671167 variant, located in intron 4, is in high linkage disequilibrium (*r* ^2^ > 0.85) with other SNPs, such as rs1903003, and is considered to tag a putative functional haplotype block (Cho et al., 2010; Chen et al., 2015). A large-scale parallel reporter assay identified physical interaction between the COPD-associated SNP rs2013701 and the FAM13A promoter, indicating that this variant modulates promoter activity and cellular proliferation. These results support a mechanism by which variant associated with both COPD and PF influence pathogenesis through FAM13A regulation (Castaldi et al., 2019).

Additional evidence indicates that the FAM13A protein interacts with SIRT1 to regulate the expression of CPT1A, the rate-limiting enzyme in mitochondrial fatty acid oxidation (FAO). The upregulation of this pathway increases the production of reactive oxygen species (ROS), thereby inducing epithelial cell death—a hallmark feature of emphysematous destruction in COPD (Eisenhut et al., 2017; Chi et al., 2006). Consequently, genetic variants that modulate FAM13A expression may influence an individual’s susceptibility to oxidative stress and cellular damage, thereby providing a mechanistic explanation for their association with progressive loss of lung function across multiple diseases (Ziółkowska-Suchanek et al., 2017; Hawkins and Mora, 2017; Jiang et al., 2017). Moreover, the expression of FAM13A is modulated by diverse environmental and cellular signals relevant to lung diseases. It is persistently upregulated under hypoxic conditions and is a documented target of the HIF-1α and TGF-β signaling pathways, thereby positioning it at the convergence of key pathways governing fibrosis, cellular stress response, and cancer. Collectively, this body of evidence delineates an integrated regulatory network that connects FAM13A expression to oxidative stress, tissue remodeling, and cellular adaptation. Such multilayered regulation provides a molecular framework to explain the phenotypic heterogeneity observed in carriers of *FAM13A* variants.

### 2.4 Part 4: disease-specific roles of FAM13A in the lung

#### 2.4.1 FAM13A in COPD

COPD represents a major global health burden, standing as the fourth leading cause of mortality worldwide with smoking established as its primary environmental risk factor (GBD 2021 Causes of Death Collaborators, 2024). The disease is characterized by persistent and progressive airflow limitation resulting from structural airway abnormalities that is not fully reversible (GBD 2021 Causes of Death Collaborators, 2024; Cornelius, 2024). However, most smokers do not develop COPD, and studies demonstrate familial clustering of the disease (Ding et al., 2015; Kauffmann et al., 1989), indicating combined contributions from genetic and environmental factors to its pathogenesis (Lee et al., 2014a). Recent GWAS have established *FAM13A* as a significant susceptibility locus associated with COPD (Cho et al., 2010).

A 2010 GWAS in Caucasian populations identified that both rs7671167 and rs1903003 were negatively associated with COPD (Cho et al., 2010). Subsequent investigations across diverse populations have consistently confirmed significant associations between *FAM13A* SNPs and COPD (Xie et al., 2015; Wang et al., 2013; Arja et al., 2014; Hancock et al., 2010; Castaldi et al., 2019; Ziółkowska-Suchanek et al., 2015; Gilowska et al., 2019; Hao et al., 2019), demonstrating the trans-ethnic generality of this genetic relationship.

Molecular analyses demonstrate that FAM13A protein expression is significantly upregulated in lung tissues of COPD smokers but shows substantial reduction after smoking cessation (Chen et al., 2023; Tam et al., 2021). Mechanistic investigations further reveal that FAM13A may contribute to COPD progression by mediating TGF-β1-induced epithelial-mesenchymal transition (EMT), thereby promoting small airway remodeling (Zhu et al., 2020). This collected evidence indicates how environmental exposures (e.g., cigarette smoke) may modulate COPD pathogenesis through regulation of FAM13A expression, suggesting the therapeutic potential of environmental interventions for respiratory diseases.

Notably, FAM13A upregulation extends beyond pulmonary tissues, with marked elevation observed in muscle tissues of COPD patients (Willis-Owen et al., 2018). Given that skeletal muscle dysfunction represents a frequent extrapulmonary manifestation whose severity correlates with overall disease progression (Seymour et al., 2010), these research outcomes suggest FAM13A’s involvement in COPD may operate through systemic mechanisms rather than being confined to the lungs.

Strength of evidence: Strong. Supported by multi-population GWAS, *in vitro* and *in vivo* functional studies, animal models, and human tissue analyses.

#### 2.4.2 FAM13A in pulmonary fibrosis

PF is a refractory and life threatening lung disease characterized by progressive and irreversible architectural destruction due to scarring, ultimately leading to organ dysfunction, impaired gas exchange, and respiratory failure (Wynn, 2011). Idiopathic pulmonary fibrosis (IPF), a particularly severe form of PF with unknown etiology, carries a 5-year survival rate below 50% (Fernández Pérez et al., 2010; King et al., 2001; Natsuizaka et al., 2014).

Genetic studies demonstrate significant associations between *FAM13A* SNPs and both IPF susceptibility and reduced survival rates (Hirano et al., 2017; Guzmán-Vargas et al., 2021). The rs2609255 variant shows particular clinical relevance, exhibiting significant associations with silicosis, rheumatoid arthritis-associated interstitial lung disease, and systemic sclerosis-related interstitial lung disease (Wang et al., 2018; Higuchi et al., 2023; Bernstein et al., 2025). Mechanistically, FAM13A overexpression has been shown to delays PF progression by suppressing M2 macrophage-derived miRNA-328, while FAM13A silencing has been associated with promoting interstitial fibroblast proliferation and exacerbates fibrosis (Yao et al., 2019). Moreover, as a modifier gene for cystic fibrosis pulmonary phenotypes, FAM13A regulates RhoA activity, actin cytoskeleton dynamics, and EMT, contributing to disease-specific lung manifestations (Corvol et al., 2018).

However, the G allele of rs2609255—a genetic risk factor associated with IPF—shows markedly different frequencies across populations: 41.5% in Asian individuals compared to only 14.4% in Hispanic white individuals. This pronounced variation underscores significant population heterogeneity in how FAM13A polymorphisms may influence susceptibility to interstitial lung disease (Hu et al., 2023), suggesting that FAM13A operates within a multifaceted regulatory framework driving disease development.

Strength of evidence: Moderate to strong. Supported by genetic associations in Asian/Hispanic populations and *in vivo* functional evidence, though European cohort data remain inconsistent.

#### 2.4.3 FAM13A in asthma

Asthma is a chronic airway inflammatory disease characterized by reversible airflow obstruction, nonspecific airway hyperresponsiveness, and persistent airway inflammation, resulting from complex interactions between polygenic inheritance and environmental factors (Guttman and Rinn, 2012). The disease demonstrates marked familial aggregation, with genetic susceptibility being particularly prominent in early childhood. Children with one asthmatic parent have a 2-5-fold increased asthma risk compared to those with non-asthmatic parents, while those with two affected parents face a tenfold higher risk (Quinn and Chang, 2016). These epidemiological patterns highlight the substantial genetic contribution to childhood asthma, necessitating focused investigation into genetic associations.

GWAS and meta-analysis in European populations revealed that the rs2869967 and rs6825998 variants are significantly associated with asthma susceptibility, suggesting their involvement in airway remodeling and inflammatory responses (Li et al., 2013). In Caucasian populations, rs987314 shows significant correlation with fractional exhaled nitric oxide levels in asthma patients (Accordini et al., 2023). At the mechanistic level, multiple studies have confirmed that the Rho-GAP domain within the protein encoded by FAM13A regulates actin cytoskeleton reorganization and intercellular junctions, thereby influencing airway smooth muscle contraction and endothelial barrier function during asthma-related airway inflammation and remodeling (Duluc and Wojciak-Stothard, 2014; Tam et al., 2021; Corvol et al., 2018; Athari, 2019).

Currently, studies on FAM13A in asthma remains predominantly focused on genetic associations without causal validation, primarily within European-ancestry populations. Mechanistic and *in vivo* functional evidence remains limited, and the cell type–specific actions of FAM13A in asthmatic airway inflammation and remodeling are yet to be clarified.

Strength of evidence: Preliminary to moderate. Primarily derived from genetic association studies in European-ancestry populations; direct functional validation in asthma-specific models is lacking.

#### 2.4.4 FAM13A in lung cancer

Lung cancer stands as a predominant contributor to global cancer incidence and mortality (Leiter et al., 2023). As with all human malignancies, its pathogenesis originates from genetic sequence alterations or dysregulated gene expression (Ziółkowska-Suchanek et al., 2017).

Genetic association studies in NSCLC cohorts indicate that the A allele of rs9224, located in the 3′untranslated region (3′UTR) of *FAM13A*, is associated with increases lung squamous cell carcinoma risk (Yu et al., 2019), whereas the rs7671167 variant appears to exert protective effects against NSCLC development (Young et al., 2011). Mechanistically, the Rho-GAP domain within the FAM13A protein functions as a pivotal regulator of tumor cell proliferation and metastasis (Eisenhut et al., 2017). FAM13A silencing has been shown to disrupts cytoskeletal architecture and consequently suppresses tumor cell proliferation and metastatic capacity (Ziółkowska-Suchanek et al., 2021). Conversely, hypoxic conditions in NSCLC cells induce FAM13A overexpression at both protein and mRNA levels, thereby promoting cellular proliferation (Ziółkowska-Suchanek et al., 2021). Collectively, these data suggest FAM13A’s context-dependent duality in lung cancer: maintaining homeostasis physiologically while potentially contributing to malignancy in tumor microenvironments.

Epidemiological investigations reveal that COPD patients carry a 2-4-fold elevated risk of developing lung cancer (Cortopassi et al., 2017). Nevertheless, the potential role of FAM13A in mediating this comorbidity relationship remains insufficiently explored.

Strength of evidence: Preliminary. Largely associative from genetic studies and observational findings; mechanistic and *in vivo* validation are needed.

**FIGURE 4:**
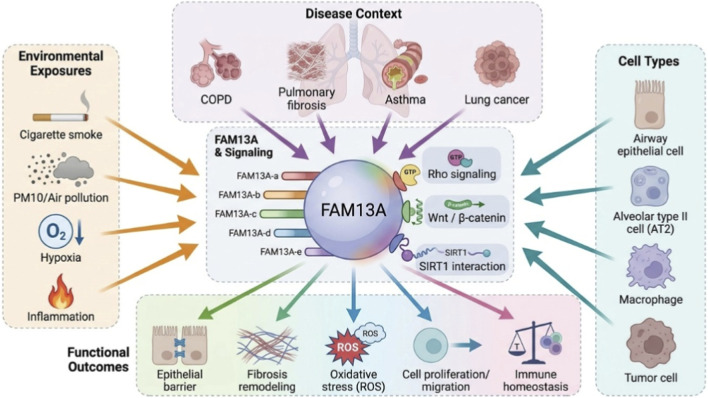
Context-dependent roles of FAM13A in pulmonary diseases. Five dimensions collectively determine the functional outcomes of FAM13A: disease type (COPD, pulmonary fibrosis, asthma, lung cancer), cell type (airway epithelial cells, alveolar type II cells, bronchial epithelial cells), environmental exposure (cigarette smoke, particulate matter, hypoxia), isoform diversity, and functional outcome (protective vs. detrimental). Take-home message: FAM13A is not intrinsically beneficial or harmful; its role varies with disease state, cell type, environmental cues, and isoform expression, reflecting its pleiotropic and context-dependent nature in pulmonary diseases.

The context-dependent roles of FAM13A across different pulmonary diseases are summarized in Figure 4, which integrates five key dimensions: disease type, cell type, environmental exposure, isoform diversity, and functional outcome.

### 2.5 Part 5. species-specific constraints in FAM13A research

Although *FAM13A* exhibits evolutionarily conserved expression patterns across species, critical divergences impose substantial research limitations. A prominent example is the long isoform (*FAM13A*-a), which contains complete functional domains and is expressed in humans and African clawed frogs, but remains undetectable in standard murine models where only truncated isoforms are observed (Howes et al., 2024) This structural distinction poses fundamental challenges for translational research, as essential domains—including the full-length RhoGAP domain strongly associated with human disease mechanisms—are missing in conventional mouse systems.

Beyond isoform variation, species-specific constraints also manifest in core physiological processes. A comparative analysis of differentially expressed genes in human COPD/emphysema and various murine models—including FAM13A^−/−^, NZW/LacJ, Hhip^+/−^, and C57BL/6—revealed remarkably limited overlap, with only two genes, P2Y14 and CHN2, shared across species. Both genes are implicated in inflammatory processes. Further interspecies divergence was observed in the regulation of antioxidant pathways. While Nrf2-regulated antioxidant genes are upregulated in the lungs of human smokers, susceptible mouse models such as A/J wild-type and ApoE^−/−^ mice exposed to chronic CS show either reduced or unchanged expression of these genes. The inflammatory regulator P2Y14 also exhibited discordant expression patterns: its expression was downregulated in human COPD patients and in the CS-resistant FAM13A^−/−^ mice, but upregulated in the CS-susceptible Hhip^+/−^ model. Moreover, when comparing genes associated with alveolar enlargement—measured by mean chord length (MCL) in mice and emphysema severity in humans—only a single gene, KAT14, was found to be consistently upregulated in both species. This collective evidence underscores the significant transcriptional disparities between human pulmonary diseases and existing mouse models of the disease (Yun et al., 2017). Moreover, varying susceptibility to CS across mouse strains highlights the additional complexity introduced by genetic background in environment–disease interactions.

In light of these multifaceted limitations, the generation of humanized FAM13A models that accurately reflect human molecular architecture and physiological traits emerges as an essential next step. Such models will be instrumental in clarifying the role of FAM13A in pulmonary disease mechanisms and advancing the development of precise therapeutic strategies.

### 2.6 Part 6. regulatory roles of the lncRNA FAM13A-AS1

The long non-coding RNA *FAM13A-AS1* is genomically adjacent to the FAM13A gene and transcribed in the opposite direction. Although it does not encode a protein, it may regulate FAM13A expression through transcriptional or post-transcriptional mechanisms (Guttman and Rinn, 2012; Wu et al., 2021). Specifically, *FAM13A-AS1* potentially modulates chromatin conformation in the FAM13A promoter region or may affect *FAM13A* mRNA stability (Quinn and Chang, 2016).

Given that FAM13A is a key player in COPD, PF, asthma, and lung cancer, understanding whether *FAM13A-AS1* regulates *FAM13A* expression in these diseases is of potential interest. However, direct evidence linking *FAM13A-AS1* to pulmonary diseases remains limited. Most current insights are derived from computational predictions or extrapolated from non-pulmonary systems. To date, no functional studies have directly investigated the role of *FAM13A-AS1* in lung disease models or patient samples.

A few observational studies have reported altered expression of *FAM13A-AS1* in certain cancers, but its specific functions in pulmonary diseases have not been characterized. Thus, whether *FAM13A-AS1* contributes to respiratory diseases through *FAM13A*-dependent or independent pathways remains an open question. Further research—particularly using loss-of-function and gain-of-function approaches in pulmonary-relevant cell types and animal models—is needed to elucidate the regulatory roles of *FAM13A-AS1* in lung health and disease.

## 3 Conclusion and perspectives

In summary, *FAM13A* serves as a pleiotropic gene with complex regulatory mechanisms that plays a critical role in maintaining pulmonary homeostasis and contributing to the pathogenesis of various lung diseases. , including COPD, PF, asthma, and lung cancer. Despite considerable research progress, the mechanistic basis of *FAM13A’s* functions remains incompletely understood, and significant interspecies expression differences—particularly the absence of the long FAM13A-a isoform in murine models—present substantial challenges for functional characterization and disease modeling.

Based on the evidence synthesized in this review, several concrete directions warrant prioritization in future research. First, regarding genetic risk stratification, rs7671167 shows consistent associations with COPD across multiple populations (European, Asian, Hispanic, and Middle Eastern) and represents a promising candidate for COPD risk assessment. The rs2609255 variant, associated with IPF susceptibility in Asian and Hispanic populations (OR = 1.47–1.81) and with silicosis risk in Chinese populations (OR = 1.71), may be useful for fibrotic lung disease risk stratification, while rs2869967 (COPD risk in Han Chinese, OR = 2.414) and rs987314 (associated with FeNO levels in asthma) warrant further evaluation as potential biomarkers, though the latter requires validation in non-European populations.

Second, patient subgroups could be stratified based on several factors. Smoking history is particularly relevant, as the genetic effects of *FAM13A* variants (e.g., rs2609264, rs2609261, rs2609260) exhibit multiplicative interactions with smoking status. Disease stage may also influence *FAM13A’s* effects, as the rs2609255 G allele accelerates DLCO decline in established IPF patients yet is associated with protection against PF development in the general population, suggesting opposing roles in early versus late disease. Additionally, hypoxic conditions, which persistently upregulate *FAM13A* expression in NSCLC cells and promote cellular proliferation, may define a subgroup of lung cancer patients with potentially more aggressive disease.

Third, among the multiple regulatory layers identified, protein stability modulation via ubiquitin-mediated degradation (triggered by AKT phosphorylation at S312) represents a particularly attractive intervention point, as this degradation facilitates proliferative repair after lung injury. Signaling pathways involving *FAM13A* are also pharmacologically targetable: the SIRT1/CPT1A/FAO axis can be modulated by CPT1A inhibitors (e.g., etomoxir), and the Wnt/β-catenin pathway has established pharmacological modulators. In contrast, targeting FAM13A splice variants or the lncRNA *FAM13A-AS1* remains more preliminary, as the functional significance of individual isoforms in pulmonary diseases has not been systematically characterized, and direct evidence linking *FAM13A-AS1* to lung diseases is lacking.

Fourth, to address the species-specific constraints imposed by the absence of the long *FAM13A*-a isoform in standard murine models, the generation of humanized *FAM13A* mice expressing the human FAM13A-a isoform is technically feasible using existing CRISPR-Cas9 knock-in strategies, given that multiple Fam13a knockout and conditional knockout models have already been successfully established. Complementary approaches, including patient-derived organoids and air-liquid interface cultures of primary human airway epithelial cells, offer platforms that retain human-specific isoform expression and bypass the interspecies divergence issues observed in conventional mouse models, such as discordant expression patterns of antioxidant genes and inflammatory regulators. iPSC-derived pulmonary cells combined with CRISPR-based gene editing provide additional platforms for investigating specific *FAM13A* variants in isogenic backgrounds relevant to individual patient subgroups.

Only through such multidisciplinary efforts—integrating humanized models, advanced cell culture systems, and targeted functional assays—can we ultimately translate discoveries on *FAM13A* into novel biomarkers and therapeutic strategies for pulmonary diseases.
